# 577 nm subthreshold micropulse laser treatment for acute central serous chorioretinopathy: a comparative study

**DOI:** 10.1186/s12886-022-02330-0

**Published:** 2022-03-05

**Authors:** He Long, Maoxiong Liu, Qinghua Hu, Xin Li

**Affiliations:** Wuhan Aier Eye Hanyang Hospital, Wu Han, China

**Keywords:** Acute central serous chorioretinopathy, 577 nm subthreshold micropulse laser, Central macular thickness, Subfoveal choroidal thickness

## Abstract

**Background:**

To assess the efficacy of 577 nm subthreshold micropulse laser (SML) treatment for acute central serous chorioretinopathy (CSC).

**Methods:**

This retrospective comparative case-series included 34 eyes of 34 patients with acute CSC who received either 577 nm SML treatment (SML group, n = 16 eyes) or were only monitored (observation group, *n* = 18 eyes). Acute CSC was defined as disease course < 3 months. Eyes with any history of treatment in the past were excluded. Data were collected over a period of 6 months. The best corrected visual acuity (BCVA), central macular thickness (CMT), and subfoveal choroidal thickness (SFCT) were observed.

**Results:**

SML group showed significantly greater improvement in the BCVA (logMAR) compared to observation group at 1 month (0.20 ± 0.10 vs 0.30 ± 0.12, *P* < 0.01), 3 months (0.13 ± 0.06 vs 0.21 ± 0.06, *P* < 0.01) and 6 months (0.01 ± 0.06 vs 0.09 ± 0.66, *P* < 0.01). The CMT reduction was significantly greater in the SML group at 1 month (337.19 ± 62.96 µm vs 395.11 ± 91.30 µm, *P* < 0.05), 3 months (312.94 ± 49.50 µm vs 364.50 ± 70.30 µm, *P* < 0.05) and 6 months (291.38 ± 26.46 µm vs 348.56 ± 54.65 µm, *P* < 0.05). In the SML group, the SFCT did not show a significant decrease at 1 month (468.88 ± 42.19 µm, *P* > 0.05) but showed a significant reduction at 3 months (451.75 ± 39.36 µm, *P* < 0.05) and 6 months (450.50 ± 34.24 µm, *P* < 0.05) from baseline (489.94 ± 45.86 µm). In the observation group, there was no significant change in SFCF during follow-up. No adverse events occurred in the SML group.

**Conclusions:**

Although some patients with acute CSC show spontaneous healing, timely intervention with 577 nm SML can shorten the disease course, improve visual acuity, and reduce the risk of chronic transformation without adverse events.

## Background

Central serous chorioretinopathy (CSC) is a disease of the retina characterized by serous detachment of the neurosensory retina, secondary to at least one focal lesion of the retinal pigment epithelium [1]. The disease most commonly affects middle-age men (mean age at presentation: 41–51 years) [2]. The identified risk factors for CSC include type A personality, psychological stress, exogenous steroid use, smoking, alcohol consumption, uncontrolled hypertension, use of psychopharmacologic medications, pregnancy, autoimmune diseases, gastroesophageal reflux, and *Helicobacter pylori* infection [3, 4]. Patients with CSC often develop reduced visual acuity, metamorphopsia, central scotoma, and loss of contrast sensitivity with progression of the disease [5]. There are two main forms of disease presentation: acute CSC and chronic CSC. Acute CSC is generally regarded as a self-limiting disease which resolves spontaneously within 3–4 months in most patients [6]; however, the reported incidence of recurrence of CSC is 33–50% [7, 8]. Cases that do not show spontaneous resolution may develop into chronic CSC, with progressive damage to the retinal pigment epithelium and photoreceptors, leading to irreversible visual impairment [9, 10].

Till date, there is no standardized treatment strategy for acute CSC [11]. Some have argued that acute CSC should only be monitored and does not require treatment [12]. However, some treatment modalities such as focal laser photocoagulation [13], photodynamic therapy (PDT) [14–16], intravitreal injection of anti-vascular endothelial growth factor (Anti-VEGF) [17], oral medication [18] and subthreshold micropulse laser (SML) [19, 20] have also been used to treat acute CSC. The clinical application of focal laser and PDT is limited owing to their serious side effects, such as central scotoma, retinal pigment epithelium atrophy, and choroidal neovascularization (CNV). According to Gawecki et al. [21], that SML should be considered in acute cases to achieve good visual results. Maruko et al. [22] and Özmert et al. [23] demonstrated the efficacy and safety of 577 nm SML for treatment of chronic CSC. However, there is a paucity of studies on the treatment of acute CSC with 577 nm SML [19, 24]. The purpose of this study was to assess the efficacy of 577 nm SML treatment for acute CSC.

## Methods

### Study design and participants

This retrospective comparative study included 34 eyes of 34 patients with acute CSC who were either treated with 577 nm SML (SML group, *n* = 16 eyes) or were only monitored (observation group, *n* = 18 eyes) at the Wuhan Aier Eye Hanyang Hospital between August 2019 and December 2020. The observation group consisted of patients with acute CSC who received no treatment during the same period. The treatment strategy was chosen by the surgeons in agreement with the patient. This study was conducted in accordance with the Declaration of Helsinki.

The diagnosis of acute CSC was confirmed by clinical examination, fundoscopy, optical coherence tomography (Cirrus HD-OCT 5000, Carl Zeiss Meditec AG, Germany), and fundus fluorescein angiography (FFA). Some patients underwent indocyanine green angiography (ICGA) and fundus autofluorescence (FAF) imaging examination (Spectralis, Heidelberg Retina Angiograph, Germany). Only eyes with acute CSC (disease duration: < 3 months) were included in this study. If both eyes were eligible for the study, the right eye was chosen in the analysis. The exclusion criteria were: (1) Acute CSC with previous history of treatment with PDT, focal photocoagulation, or intravitreal anti-VEGF injections; (2) systemic steroid therapy; (3) other retinal diseases such as pathological myopia, CNV, polypoidal choroidal vasculopathy (PCV), or history of intraocular surgery; (4) Incomplete follow-up and case data.

### Data collection

All patients were examined at baseline and at 1 month, 3 months, and 6 months after treatment, including slit-lamp biomicroscopy, dilated fundus examination, intraocular pressure measurements, best corrected visual acuity (BCVA), and spectral- domain optical coherence tomography (SD-OCT). FFA was performed at baseline and repeated as needed. ICGA and FAF results were available for 56% and 41% patients, respectively. The BCVA was measured with a standard visual acuity chart, and the decimal BCVA was converted to the logarithm of the minimum angle of resolution (logMAR) units for the statistical analyses. Central macular thickness (CMT) was defined as the distance between the internal limiting membrane and the inner border of the RPE by OCT [19, 25], which included the subretinal fluid (SRF), if present. Using a virtual caliper the subfoveal choroidal thickness (SFCT) was measured from enhanced depth imaging (EDI) scan by taking the vertical distance between the hyper-reflective line of Bruch’s membrane and the innermost hyper-reflective line of the choroid scleral interface, and the average of the two scans (vertical and horizontal) was regarded as the SFCT [26].

### 577 nm micropulse laser treatment

The pupil was dilated with 0.5% tropicamide phenylephrine eye drops (Mydrin-P, Santian Pharmaceutical Co. Ltd., China). Then, 0.4% oxybuprocaine hydrochloride eye drops (Benoxil, Santian Pharmaceutical Co. Ltd., China) were used for superficial anesthesia. Iridex IQ 577 nm SML treatment (IQ 577; IRIDEX, America) was performed by the same experienced surgeon (Xin Li). The parameters for 577 nm SML treatment were: spot diameter, 200 μm; exposure time, 200 ms; duty cycle, 5%; TxCell fusion laser matrix, 7 × 7; laser spot spacing, 0. Energy titration was performed using a continuous wavelength laser in the peripapillary nasal 2DD area. The initial titration energy was 50 mW, and the energy was gradually increased until the appearance of the retinal spot (light gray spot of grade I). Quadruple of the titration energy is the treatment energy of the SML. Then, the SML multi-point mode was used to perform full-coverage laser photocoagulation therapy on the serous retinal detachment area. The number of laser envelopes, laser power energy, and photocoagulation sessions were recorded. At one month review, the same experienced surgeon (Xin Li) decided whether or not to repeat SML therapy. The criterion for repeat treatment was the presence of SRF in the macular area, and the criteria for termination of treatment were the complete absorption of SRF or completion of three treatments. Complete resolution was said to have been achieved if the macula showed no SRF on OCT images.

### Statistical analyses

The primary outcome measure was the change in the BCVA on follow-up. Secondary outcome measures were change in CMT and SFCT at 6 months. The BCVA was converted to the logarithm of the minimum angle of resolution (logMAR) units for statistical analyses. Statistical analysis included comparison of the baseline data and follow-up data in each group. All statistical analyses were performed using SPSS software (version 23.0, IBM SPSS). Continuous variables were presented as mean ± standard deviation and between-group differences assessed using independent *t* tests (parametric data distribution). Levene test was used to test homogeneity of variance. Fisher test was used to compare categorical data between the two groups. Two tailed *P* values < 0.05 were considered indicative of statistical significance.

## Results

### Baseline demographic and clinical characteristics

Thirty-four patients (23 men, 11 women) were enrolled in this study. The mean age of patients was 40.47 ± 6.77 years. There was no significant difference between the SML group and observation group with respect to the mean duration of symptoms until baseline visit (6.99 ± 1.75 days versus 6.54 ± 1.54 days; *P* = 0.306). The mean number of 577 nm SML treatment sessions in the SML group was 2.0 ± 0.82. At baseline, there were no significant between-group differences with respect to sex, age, duration of symptoms, BCVA (logMAR), CMT, or SFCT (*P* > 0.05 for all) (Table 1).

**Table 1:** Baseline demographic and clinical characteristics of the study population

	Micropulse Laser (*n* = 16)	Observation (*n* = 18)	*P* value
Sex, male (n)	12	11	0.388
Age (years, mean ± SD)	41 ± 7.47	40 ± 6.25	0.677
Duration of symptoms (days, mean ± SD)	6.99 ± 1.75	6.54 ± 1.54	0.306
Number of laser treatments	2.0 ± 0.82		
BCVA (logMAR, mean ± SD)	0.48 ± 0.20	0.56 ± 0.22	0.310
CMT (µm, mean ± SD)	485.38 ± 151.44	483.50 ± 110.89	0.967
SFCT (µm, mean ± SD)	489.94 ± 45.86	493.78 ± 44.02	0.805

*BCVA* best corrected visual acuity; *CMT* central macular thickness, *logMAR* logarithm of the minimum angle of resolution; *SFCT* subfoveal choroidal thickness; *SD* standard deviation

### Changes in visual acuity

**Fig.1:**
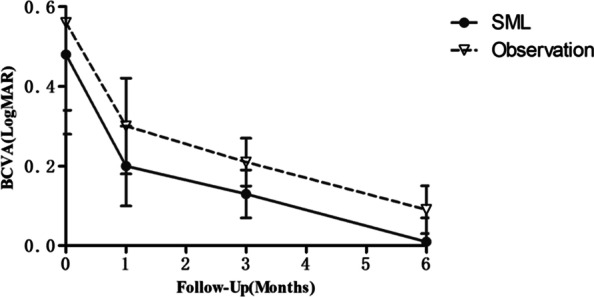
Changes in the mean best corrected visual acuity (BCVA) from baseline to 6 months in the two groups. LogMAR, logarithm of the minimum angle of resolution; SML, subthreshold micropulse laser

The changes in BCVA (LogMAR) at different time-points are shown in Fig. 1. In the 577 nm SML group, BCVA (logMAR) improved significantly from 0.48 ± 0.20 at baseline to 0.20 ± 0.10 at 1 month, 0.13 ± 0.06 at 3 months, and 0.01 ± 0.06 at 6 months (*P* < 0.01). Significant improvement in the BCVA (logMAR) was also observed in the observation group from baseline (0.56 ± 0.22) to 1 month, 3 months, and 6 months (0.30 ± 0.12, 0.21 ± 0.06, 0.09 ± 0.06, respectively) (*P* < 0.01). Improvement in visual acuity was more significant in the SML group than in the observation group (0.20 ± 0.10 vs 0.30 ± 0.12, 0.13 ± 0.06 vs 0.21 ± 0.06, 0.01 ± 0.06 vs 0.09 ± 0.06, respectively) (*P* < 0.05).

### Changes in central macular thickness

**Fig.2:**
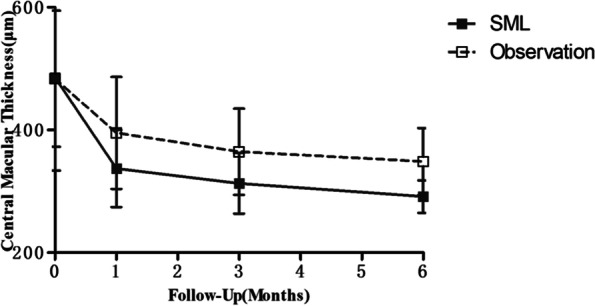
Changes in the mean central macular thickness from baseline to 6 months in the two groups. SML, subthreshold micropulse laser

In the 577 nm SML group, mean CMT decreased from 485.38 ± 151.44 µm at baseline to 337.19 ± 62.96 µm at 1 month, 312.94 ± 49.50 µm at 3 months, and 291.38 ± 26.46 µm 6 months *(P* < 0.01) (Fig. 2). In the observation group, mean CMT was 483.50 ± 110.89 µm at baseline, 395.11 ± 91.30 µm at 1 month, 364.50 ± 70.30 µm at 3 months, and 348.56 ± 54.65 µm at 6 months (*P* < 0.05) (Fig. 2). During follow-up, compared with the observation group, the SML group showed a more obvious decrease in CMT, and the difference was statistically significant (*P* < 0.05).

### Changes in subfoveal choroidal thickness

**Fig.3:**
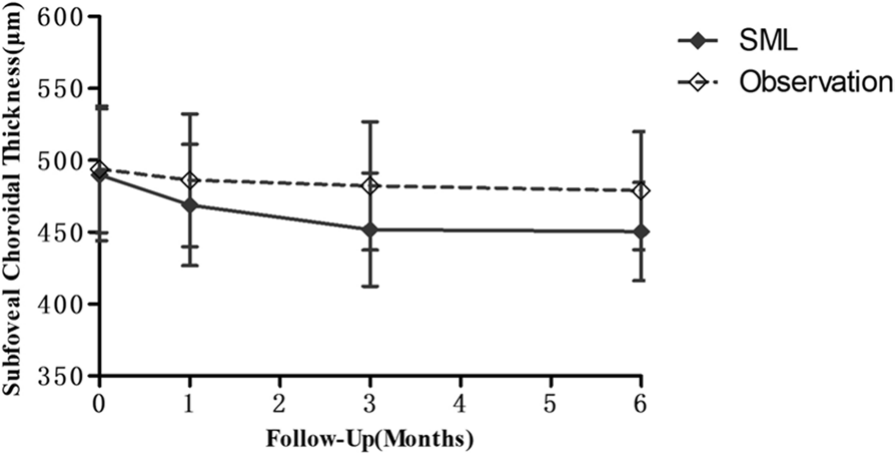
Changes in the mean subfoveal choroidal thickness from baseline to 6 months in the two groups. SML, subthreshold micropulse laser

In the SML group, the mean SFCT decreased from 489.94 ± 45.86 µm at baseline to 468.88 ± 42.19 µm at 1 month (*P* = 0.186), 451.75 ± 39.36 µm at 3 months (*P* = 0.017), and 450.50 ± 34.24 µm at 6 months (*P* = 0.10) (Fig. 3). In the observation group, the mean SFCT was 493.78 ± 44.02 µm at baseline, 486.06 ± 46.12 µm at 1 month (*P* = 0.611), 482.22 ± 44.64 µm at 3 months (*P* = 0.440), and 478.78 ± 41.02 µm at 6 months (*P* = 0.298) (Fig. 3). The decrease in SFCT in the observation group was not statistically significant. In the 577 nm SML group, the reduction in SFCT was significant at 3 months (*P* = 0.044) and 6 months (*P* = 0.038), compared with the observation group.

### Safety

**Fig.4:**
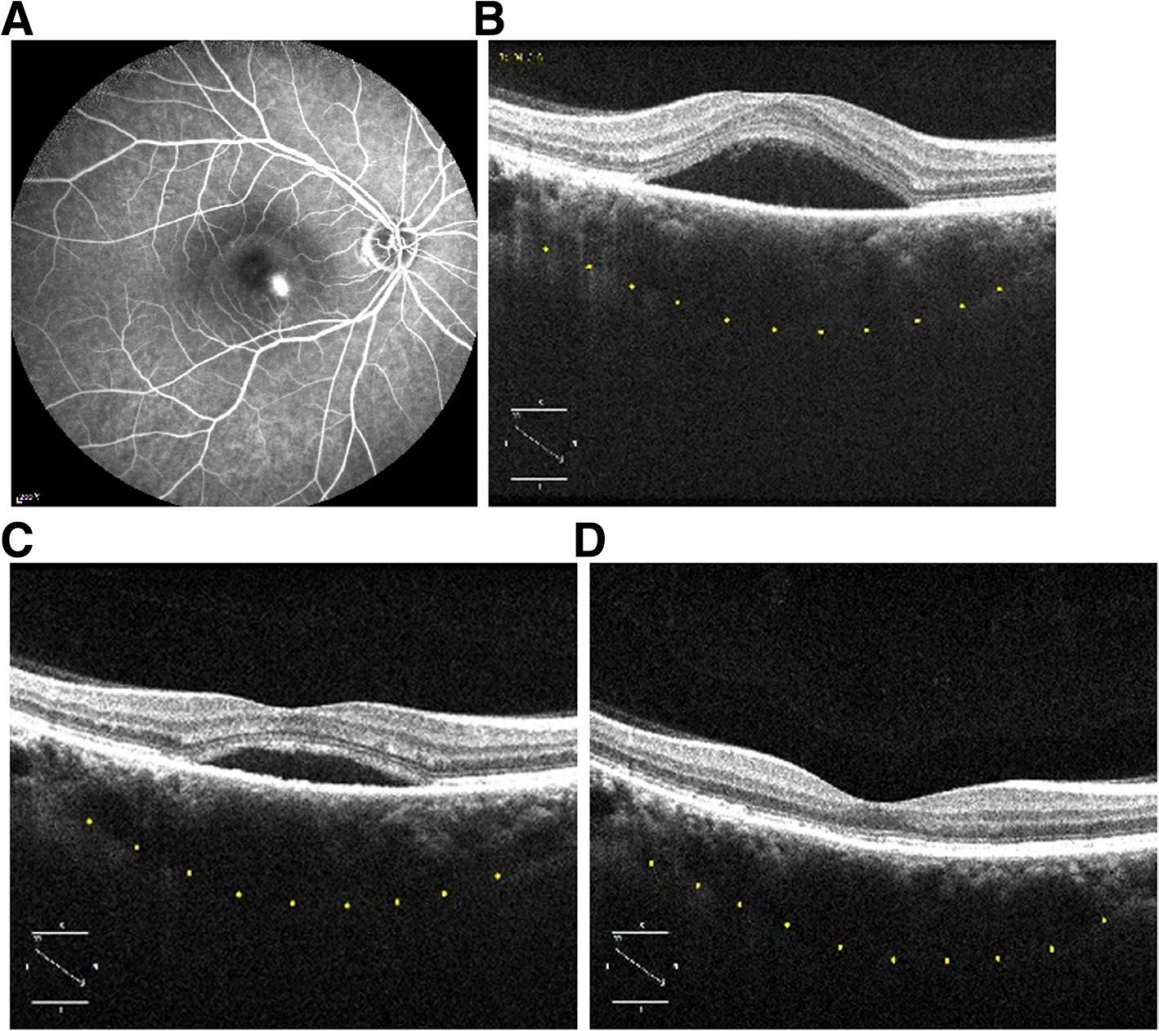
Fundus fluorescein angiography (FFA) and optical coherence tomography (OCT) of a patient with acute CSC at baseline, 1 month, and 3 months. A) A hot spot of leakage is visible on FFA at baseline. B and C show subretinal fluid (SRF) on OCT at baseline and 1 month after SML treatment, respectively. D) The SRF has completely resolved 3 months after SML treatment; no signs of laser-induced damage are seen

No structural changes in RPE, photoreceptor layer, or inner and outer retinal layers were observed on fundoscopy, OCT imaging, or on FFA at the end of follow-up (Fig. 4).

## Discussion

CSC is the fourth most frequent cause of retinopathy, after age-related macular degeneration, diabetic retinopathy, and retinal vein occlusion [27], and the condition is usually classified as acute or chronic. Most episodes of CSC are self-limiting and typically show spontaneous resolution within 3 to 4 months [28]; therefore, it was generally believed that patients with acute CSC should be initially monitored for at least 3 months before considering treatment. However, up to 50% patients with CSC may develop recurrence and in up to 20% patients, the disease becomes chronic [29, 30]. Recurrent episodes of CSC, persistent SRF and/or pigment epithelium detachment, and RPE atrophy may lead to permanent visual impairment [7]. Scholz et al. [31] suggested that patients should be treated as early as possible while they are still in the acute stage in order to prevent any permanent structural damage. In our study, to differentiate the efficacy of 577 nm SML from the natural course of the disease, we enrolled a control group of patients with acute CSC during the same time-period who received no treatment. From baseline to 6-month follow-up, the 577 nm SML group showed better BCVA (logMAR) compared to the observation group, and the CMT also decreased more obviously. Similar results were reported by Zhou et al. [19] and Arora et al. [20] Zhou et al. [19]. reported that at 3 months, 577 nm SML with 50% threshold power and 25% threshold power improved BCVA (logMAR) and reduced CMT compared to baseline in patients with acute CSC. In the study by Arora et al. [20]. 810 nm SML group showed significantly higher BCVA (logMRA) at 2, 4, 8, and 16 weeks, and six months compared with the observation group for acute CSC. During follow-up, the changes in contrast sensitivity, the height of neurosensory detachment, and the width of the neurosensory detachment in the 810 nm SML group were better than those in the observation group. 577 nm SML is a quite different modality than 810 nm SML. The 577 nm SML is absorbed by both melanin and oxyhemoglobin, which leads to maximum absorption in the pigment epithelium and choriocapillaris [32], and minimizes the absorption of yellow light by lutein in the inner and outer cluster layers of macula; therefore, the treatment of macular fovea and its vicinity is relatively safe [33].

In addition, our study also showed a decrease in SFCT beginning at 3 months after 577 nm SML treatment. However, there was no significant change in SFCT in the observation group. In the study by Rocal et al. [34], 577 nm SML and half-dose PDT treatment for chronic CSC significantly diminished SFCT. Sun et al. [35] also observed a significant reduction in the mean SFCT from baseline to week 12 in the SML group and in the threshold conventional laser group. Acute CSC has a short disease course of approximately 3 months and does not result in any irreversible structural damage; spontaneous healing over time results in reduced choroidal vascular permeability and recovery of choroidal thickness. Since acute CSC spontaneously heals by approximately 3 months, the decrease in SFCT at 6 months should be the result of 577 nm SML therapy rather than spontaneous absorption. However, we found that the amplitude of SFCT reduction in the 577 nm SML group was small, which may be related to the fact that SML mainly acts on the RPE.

The mechanism by which SML therapy induces changes in SFCT is unclear. According to Arora et al. [20], 810 nm SML causes resolution of CSC by normalization of RPE function and retinal autoregulation by means of sublethal RPE heat-shock protein activation or possibly by causing cytokine expression which decreases choroidal hyper-permeability. We believe that the decrease in SFCT is attributable to the following reasons: CSC is a pachychoroid disease characterized by localized or diffuse choroidal thickening and/or dilatation of the outer choroidal vessels (Haller layer), atrophy of the inner choroidal capillary layer and sattler layer, damage to RPE, and hyperpermeability of the choroidal vessels. A previous study documented a decrease in SFCT after therapy, further illustrating the role of choroidal vasculature in the pathogenesis of CSC [36–39]. SML targets RPE and exerts heat stress on RPE cells, eliciting their biological response rather than causing cell death. Heating of the RPE complex with high-density light spots induces the expression of HSP70, inhibits cell apoptosis, and down-regulates the expression of inflammatory factors. At the same time, stimulation of sublethal RPE cells by light induces the production of pigment epithelial-derived factors and VEGF inhibitory factors, restoring the pumping function and external barrier. These changes reduce the choroidal permeability to achieve the therapeutic effect [20, 33, 40, 41].

Finally, in our study, no adverse effects were noted during or after 577 nm SML treatment, and none of the patients reported any adverse effects related to 577 nm SML. Our results are consistent with those of Maruko et al. [22] and Özmert et al. [23] who used 577 nm SML to treat chronic CSC. Although focal laser [13], PDT [14–16] and intravitreal injection Anti-VEGF [17] have been shown to be effective in the treatment of acute CSC, focal laser may lead to permanent scotoma, which enlarges over time with RPE scar expansion, and may potentially cause choroidal neovascularization [42]. PDT may also cause several adverse effects such as RPE atrophy, choroidal ischemia and secondary CNV [43, 44]. In addition, PDT is expensive and the lack of relevant drugs in China is a barrier to its wider use. Anti-VEGF agents have shown promising results only in the subgroup of patients with CNVs secondary to CSC [45]; in addition, the treatment is costly and requires repeated intravitreal injection, which affects patient compliance. Therefore, 577 nm SML would be an effective and safe alternative therapy for acute CSC.

Some potential limitations of our study should be acknowledged. First, the study was a retrospective study with a small sample size and a relatively short follow-up period. Second, the conversion of decimal BCVA to logMAR units may lead to overestimation of its true value according to a previous report [46], especially in patients with poorer acuity. Thus, the significant improvement in BCVA (logMAR) in the 577 nm SML group should be interpreted with caution. Third, Tan et al. [47] demonstrated a significant and consistent diurnal variation in SFCT in normal individuals. SFCT measurements in this study did not take into account the time of day at which the OCT scan was performed, which requires further study. In addition, we measured the SFCT manually, which may affect the accuracy of measurement. In some patients, the scleral border was insufficiently detectable on EDI-OCT imaging, which may have hindered SFCT analysis.

In conclusion, this retrospective study demonstrated the efficacy of 577 nm SML as a treatment option for acute CSC. No adverse events related to 577 nm SML were observed in this cohort. Prompt treatment of acute CSC with a 577 nm SML may result in better visual outcomes. Larger, prospective, randomized controlled clinical trials with longer follow-up are required to provide more robust evidence.

## Data Availability

The datasets used and/or analysed during the current study are available from the corresponding author on reasonable request.

## Abbreviations

CSC: Central serous chorioretinopathy
RPE: Retinal pigment epithelial
PDT: Photodynamic therapy
Anti-VEGF: Anti-vascular endothelial growth factor
SML: Subthreshold micropulse laser
CNV: Choroidal neovascularization
FFA: Fundus fluorescein angiography
ICGA: Indocyanine green angiography
FAF: Fundus autofluorescence
PCV: Polypoidal choroidal vasculopathy
BCVA: Best corrected visual acuity
SD-OCT: Spectral-domain optical coherence tomography
logMAR: Logarithm of the minimum angle of resolution
CMT: Central macular thickness
SFCT: Subfoveal choroidal thickness
SRF: Subretinal fluid
EDI: Enhanced depth imaging
